# Cardiac late effects after modern 3D-conformal radiotherapy in breast cancer patients: a retrospective cohort study in Germany (ESCaRa)

**DOI:** 10.1007/s10549-021-06412-3

**Published:** 2021-10-09

**Authors:** Hiltrud Merzenich, Dan Baaken, Marcus Schmidt, Inga Bekes, Lukas Schwentner, Wolfgang Janni, Achim Woeckel, Detlef Bartkowiak, Thomas Wiegel, Maria Blettner, Daniel Wollschläger, Heinz Schmidberger

**Affiliations:** 1 University Medical Center Mainz, Institute of Medical Biostatistics, Epidemiology and Informatics, 55101 Mainz, Germany; 2 Department of Obstetrics and Gynecology, University Medical Center Mainz, 55101 Mainz, Germany; 3 Department of Gynecology and Obstetrics, University Hospital Ulm, Prittwitzstr. 43, 89075 Ulm, Germany; 4 University Hospital Würzburg, Josef-Schneider-Straße 4, 97080 Würzburg, Germany; 5 Department of Radiation Oncology, University Hospital Ulm, Albert-Einstein-Allee 23, 89081 Ulm, Germany; 6 Department of Radiation Oncology and Radiation Therapy, University Medical Center Mainz, 55101 Mainz, Germany

**Keywords:** Breast cancer, 3D-conformal radiotherapy, Cardiac mortality, Cardiac morbidity, Cohort study, Survival

## Abstract

**Purpose:**

Radiotherapy (RT) was identified as a risk factor for long-term cardiac effects in breast cancer patients treated until the 1990s. However, modern techniques reduce radiation exposure of the heart, but some exposure remains unavoidable. In a retrospective cohort study, we investigated cardiac mortality and morbidity of breast cancer survivors treated with recent RT in Germany.

**Methods:**

A total of 11,982 breast cancer patients treated between 1998 and 2008 were included. A mortality follow-up was conducted until 06/2018. In order to assess cardiac morbidity occurring after breast cancer treatment, a questionnaire was sent out in 2014 and 2019. The effect of breast cancer laterality on cardiac mortality and morbidity was investigated as a proxy for radiation exposure. We used Cox Proportional Hazards regression analysis, taking potential confounders into account.

**Results:**

After a median follow-up time of 11.1 years, there was no significant association of tumor laterality with cardiac mortality in irradiated patients (hazard ratio (HR) for left-sided versus right-sided tumor 1.09; 95% confidence interval (CI) 0.85–1.41). Furthermore, tumor laterality was not identified as a significant risk factor for cardiac morbidity (HR = 1.05; 95%CI 0.88–1.25).

**Conclusions:**

Even though RT for left-sided breast cancer on average incurs higher radiation dose to the heart than RT for right-sided tumors, we found no evidence that laterality is a strong risk factor for cardiac disease after contemporary RT. However, larger sample sizes, longer follow-up, detailed information on individual risk factors and heart dose are needed to assess clinically manifest late effects of current cancer therapy.

**Supplementary Information:**

The online version contains supplementary material available at 10.1007/s10549-021-06412-3.

## Introduction

Annually more than two million women are diagnosed with breast cancer [1]. The prognosis of breast cancer has improved substantially. Currently the relative 5-year survival exceeds 80% in many countries [2], partly due to earlier diagnosis and adjuvant therapies. Radiotherapy (RT) is an important component of breast cancer treatment and reduces local recurrence and breast cancer mortality after breast conserving surgery [3]. Despite the benefit, long-term cardiac side effects related to RT are of clinical concern. Radiation-induced cardiotoxicity is caused by lesions of the microvasculature or by conduction abnormalities and arrhythmias related to autonomic dysfunction [4–6].

Observational studies have shown that breast cancer patients with RT for left-sided tumors had higher risk for coronary heart disease and cardiac mortality compared to those with right-sided RT [7]. A review [8] based on studies from 28 countries reported a mean dose to the whole heart of 5.4 Gray (Gy) on average for left-sided breast cancer and 3.3 Gy for right-sided breast cancer. Assuming that tumor laterality is almost completely random, observational studies comparing cardiac outcomes in women irradiated for left- versus right-sided breast cancer may reveal the effect of higher vs. lower radiation doses [9].

Clinical manifestations of cardiac outcomes after RT in breast cancer patients seem to decrease with each decade [7, 10]. Beginning in the late 1980s [11], advances in RT including techniques such as 3D-treatment planning, and respiratory gating have substantially reduced cardiac dose. However, even modern RT may cause a cardiac risk [12] since the heart remains exposed, mainly depending on tumor laterality and individual anatomical risk factors [8, 12]. Studies with detailed information about individual cardiac dose have shown a linear dose–response relationship [13, 14].

Assessing the long-term cardiac risk of patients treated with modern RT requires decades of follow-up. The risk for coronary heart disease started to increase within the first decade after breast cancer and continued into the following decades [13]. With a low number of events and the competing mortality risk from breast cancer, observational studies require large sample sizes [10]. Furthermore, accounting for individual patient risk factors requires detailed data on cardiovascular comorbidity. Breast cancer patients can also receive anthracyclines and other cardiotoxic drugs whose effects on cardiovascular outcomes are difficult to disentangle from those of RT [5].

The presented ESCaRa-Study (Epidemiological Study on Cardiac late effects and second malignancies after Radiotherapy in breast cancer patients) focused on cardiac mortality and cardiac morbidity in breast cancer patients in Germany treated between 1998 and 2008 with contemporary RT. ESCaRa continues the PASSOS-Heart Study [15, 16] with additional 5.5 years of systematic mortality follow-up and a second questionnaire survey on cardiac morbidity in 2019.

## Methods

### Study design and study population

The ESCaRa-study is a retrospective multicenter cohort study. Eligible breast cancer patients were diagnosed and treated between 01/1998 and 12/2008 at Mainz University Medical Center, at the Ulm University Hospital or at one of 16 partner clinics in the vicinity of Ulm. Inclusion criteria were a histologically confirmed primary and loco-regional breast cancer disease, either an invasive carcinoma or a carcinoma in situ (CIS). Since the focus was on breast cancer patients with a good prognosis, women with primary metastatic disease or bilateral breast cancer were not included.

Individual data on breast cancer disease, therapy and co-morbidities were abstracted from the patients’ hospital records: date of diagnosis, age at diagnosis, date of birth, laterality, TNM-stage, histological subtype, grading, lymphatic and vascular invasion, and hormonal parameters. Treatment information included type of surgery, adjuvant systemic chemotherapy, adjuvant endocrine therapy, and detailed information on administered adjuvant RT. Data on first recurrences as well as on second primary tumors were obtained. Furthermore, the history of malignant diseases prior to breast cancer was abstracted.

Cardiac morbidities diagnosed at the time of breast cancer treatment were recorded, if available from the pre-anesthetic interview documentation: New York Heart Association (NYHA) cardiac score at the time of surgery, history of myocardial infarction, coronary heart disease, angina pectoris, dysrhythmia, vitium cordis, stroke, and pacemaker.

### Mortality follow-up and mortality endpoints

The individual follow-up was carried out by contacting the corresponding compulsory population registry of the municipality of the last known residence in order to assess the vital status on June 30, 2018. Death certificates were obtained from the local health authority of the place of death. The underlying causes of death were coded according to the 10th revision of the International Classification of Diseases (ICD).

The definition of mortality endpoints was adapted from [17]. The primary mortality endpoint is defined as all deaths certified as heart disease (cardiac infarction I21–I23; chronic ischemic heart disease I25.0–I25.9; acute ischemic heart disease I21.0–I24.9; congestive heart failure I50.0–I50.9; angina pectoris I20.0–I20.9; cardiac arrest I46; dysrhythmias, conduction disorder I44.0–I49.9; vitium cordis I34.0–I37.9). Additional analyses were carried out for all-cause mortality (A00-Y98).

### Questionnaire survey and morbidity endpoints

In 2014, a questionnaire was mailed to former patients who were reported alive. It contained 25 questions on sociodemographic variables, clinical diagnoses of cardiac illness before or after cancer treatment, potential risk factors for cardiac disease, the duration of hormonal therapy, and about cancer recurrence. Patients who did not wish to complete the extensive questionnaire could fill out a short form with questions on cardiac illness and on cardiovascular risk factors (supplementary information: SI 1). In 2019, a second questionnaire survey was conducted: the short form was sent out to patients who (1) participated in the first survey, (2) who were reported to be alive and (3) who gave permission for a second contact.

An incident cardiac event was defined to be present if a participant reported any of the following individual events after breast cancer therapy: Myocardial infarction, angina pectoris, congestive heart failure, arrhythmia, or valvular heart disease [17].

### RT techniques

The patients underwent RT at the University Medical Centers in Mainz, Ulm or at one of 16 collaborative partner clinics, who had their own RT unit. The whole breast was treated by three-dimensional conformal RT using two tangential fields of 6 MV photons. The total radiation dose to the planning target volume typically was 50 Gy, administered in 25 fractions with 5 fractions per week. For breast conserving treatment, an additional boost dose of 10 Gy was usually delivered to the tumor bed. Optionally, RT could include a supraclavicular field as well as an anterior–posterior parasternal field to cover the internal mammary lymph nodes [18, 19]. Active breathing modalities or altered fractionation were not applied in this cohort.

### Statistical methods

Individual follow-up (time at risk) started with the date of diagnosis of primary breast cancer. We included all diagnoses occurring since January 1, 1998. The end of the mortality follow-up was defined as the date of death, last information date, or June 30, 2018, whichever occurred first. The questionnaire-based assessment of morbidity included all self-reported diagnoses occurring since January 1, 1998. For patients who responded to the questionnaire, the end of follow-up was defined as the event date (reported year of cardiac diagnosis), last information date, or June 30, 2018, whichever occurred first.

Missing information on chemotherapy, endocrine therapy and body mass index (BMI) at diagnosis was imputed via multiple imputation using fully conditional specification method, assuming data missing at random. We used SAS procedures PROC MI and PROC MIANALYZE to create 20 imputed datasets, and pooled individual modeling results based on the imputed datasets using Rubin’s Rule. Complete case analyses were conducted as sensitivity analysis [20–23].

For patients who received RT, the Kaplan–Meier Method was used to assess survival. Survival of patients with left- vs. right-sided RT was compared by the Mantel–Haenszel (log-rank) test [24]. Multivariable Cox-Regression was used to assess the association of cardiac events with breast cancer laterality as a surrogate measure for the level of radiation exposure [25]. Hazard ratios (HR) and corresponding 95%-confidence intervals (CI) were estimated. The significance level was set at 5% without correction for multiple testing. The analysis was adjusted for potential confounders selected a-priori based on theoretical considerations (age at breast cancer diagnosis, year of breast cancer diagnosis, baseline cardiac morbidity (yes/no), application of chemotherapy (yes/no), application of endocrine therapy (yes/no), and BMI). To allow for a potentially non-monotonic association of BMI with cardiac endpoints, BMI was included as a cubic spline with four internal knots. The analysis was restricted to patients with at least 1 year of follow-up, and only cardiac deaths that occurred at least 1 year after breast cancer diagnosis. Incident cardiac events were only considered if the reported calendar year was later than the year of breast cancer diagnosis. If women participated in both surveys, the first morbidity event was considered for the analysis. The proportional hazards assumption was checked by examining complementary log–log plots for categorical variables, and by assessing the correlation of scaled Schoenfeld residuals with log survival time for continuous variables. For a sensitivity analysis, data were censored at time of diagnosis of a recurrent event since additional RT or chemotherapy may have occurred thereafter.

SAS for windows, version 9.4 (SAS Institute Inc., Cary, North Carolina) was used for all analyses.

## Results

### Description of the cohort at baseline

A total of 11,982 women met the inclusion criteria. Most patients were diagnosed after 2000 (Table 1). The mean age at diagnosis was 60.9 years (range 18–101 years). A total of 2925 (24.4%) patients received no RT. These women were older on average (mean age 66.7 years) compared to patients who were treated with RT (mean age 59.0 years). Patients who did not receive RT had a higher percentage of CIS compared to irradiated patients. This is consistent with 67.4% of women in this group having had mastectomy without adjuvant chemotherapy and a higher frequency of previous cardiac disease compared to irradiated women. More than 75% of the patients (*n* = 9.057) received RT as part of the primary treatment. Proportions of women with left-sided or right-sided breast cancer among those with RT were similar. The distribution of age at diagnosis, calendar year of diagnosis, staging, the application of chemotherapy, endocrine therapy and the type of surgery were similar for left- and right-sided tumors. A history of cardiac disease was confirmed in approximately 8% in both laterality groups.

**Table 1:** Characteristics of the study population by radiotherapy group

	No radiation therapy	Yes radiation therapy	
		Right-sided	Left-sided
Year of diagnosis			
1998–2000	210 (7.2%)	321 (7.2%)	334 (7.2%)
2001–2003	604 (20.6%)	710 (16.0%)	786 (17.0%)
2004–2006	1272 (43.5%)	1864 (42.1%)	1912 (41.4%)
2007–2008	839 (28.7%)	1539 (34.7%)	1591 (34.4%)
Age at diagnosis (years)			
Mean	66.7	58.7	59.3
SD*	15.5	12.3	12.0
T-stage (*N*, %)			
1	955 (32.6%)	2458 (55.4%)	2568 (55.6%)
2	986 (33.7%)	1427 (32.2%)	1454 (31.4%)
3	114 (3.9%)	151 (3.4%)	191 (4.1%)
4	188 (6.5%)	167 (3.8%)	174 (3.8%)
In situ	548 (18.7%)	176 (3.9%)	198 (4.3%)
Unknown	133 (4.6%)	55 (1.2%)	38 (0.8%)
N-stage (*N*, %)			
0	1477 (50.5%)	2633 (59.4%)	2,738 (59.2%)
1	585 (20.0%)	1065 (24.0%)	1,094 (23.7%)
2	153 (5.2%)	380 (8.6%)	378 (8.2%)
3	76 (2.6%)	228 (5.1%)	244 (5.3%)
X	634 (21.7%)	128 (2.9%)	169 (3.6%)
Chemotherapy (*N*, %)			
Yes	606 (20.7%)	2,254 (50.8%)	2,266 (49.0%)
No	2309 (78.9%)	2140 (48.3%)	2323 (50.3%)
Unknown	10 (0.4%)	40 (0.9%)	34 (0.7%)
Endocrine therapy (*N*, %)			
Yes	1604 (54.8%)	3273 (73.8%)	3411 (73.8%)
No	1243 (42.5%)	1012 (22.8%)	1070 (23.1%)
Unknown	78 (2.7%)	149 (3.4%)	142 (3.1%)
Type of surgery			
None	82 (2.8%)	16 (0.4%)	14 (0.3%)
Breast conserving	851 (29.1%)	3706 (83.6%)	3837 (83.0%)
Mastectomy	1971 (67.4%)	710 (16.0%)	772 (16.7%)
Unknown	21 (0.7%)	2 (0.05%)	0 (0.0%)
History of cardiac disease^a^ (*N*, %)			
Yes	355 (12.1%)	383 (8.6%)	371 (8.0%)
No information	2570 (87.9%)	4051 (91.4%)	4252 (92.0%)
BMI^b^ at diagnosis			
< 18.5	65 (2.2%)	69 (1.6%)	58 (1.3%)
18.5–24.9	1187 (40.6%)	1810 (40.8%)	1877 (40.6%)
25.0–29.9	801 (27.4%)	1364 (30.8%)	1426 (30.8%)
≥ 30.0	454 (15.5%)	816 (18.4%)	869 (18.8%)
No information	418 (14.3%)	375 (8.5%)	393 (8.5%)
Total *N* = 11,982	2925 (100%)	4434 (100%)	4623 (100%)

*SD standard deviation

^a^History of cardiac disease at the time of breast cancer diagnosis: cardiac infarction or coronary heart disease or angina pectoris or NYHA ≥ 3 or dysrhythmia or vitium cordis or pacemaker

^b^*BMI* body mass index

In a randomly selected sample of 769 irradiated breast cancer patients, electronic treatment planning records were obtained. The average mean heart dose (median) was 4.6 Gy (3.7 Gy) for left-sided RT, and 1.7 Gy (1.4 Gy) for right-sided RT [18].

### Mortality follow-up

Vital status of the cohort was observed until June 30, 2018 yielding a median follow-up period of 11.1 years. At the end of the follow-up period, more than 65% of the former breast cancer patients were still alive (Table 2)*.* Among those women who did not receive RT, the proportion of deaths was 51.8% compared to 27.9% in women with RT. For *n* = 126 (3%) of deceased cohort members, no cause of death could be ascertained.

**Table 2:** Overall survival of the ESCaRa-cohort (end of observation June 30, 2018)

	Radiotherapy no (*n*, %)	Radiotherapy yes (*n*, %)	All (*N*)
Alive	1382 (47.3%)	6435 (71.1%)	7817 (65.2%)
Dead	1514 (51.7%)	2523 (27.9%)	4037 (33.7%)
Lost to follow-up	29 (1.0%)	99 (1.0%)	128 (1.1%)
Total	2925 (100%)	9057 (100%)	11,982 (100%)

**Fig. 1:**
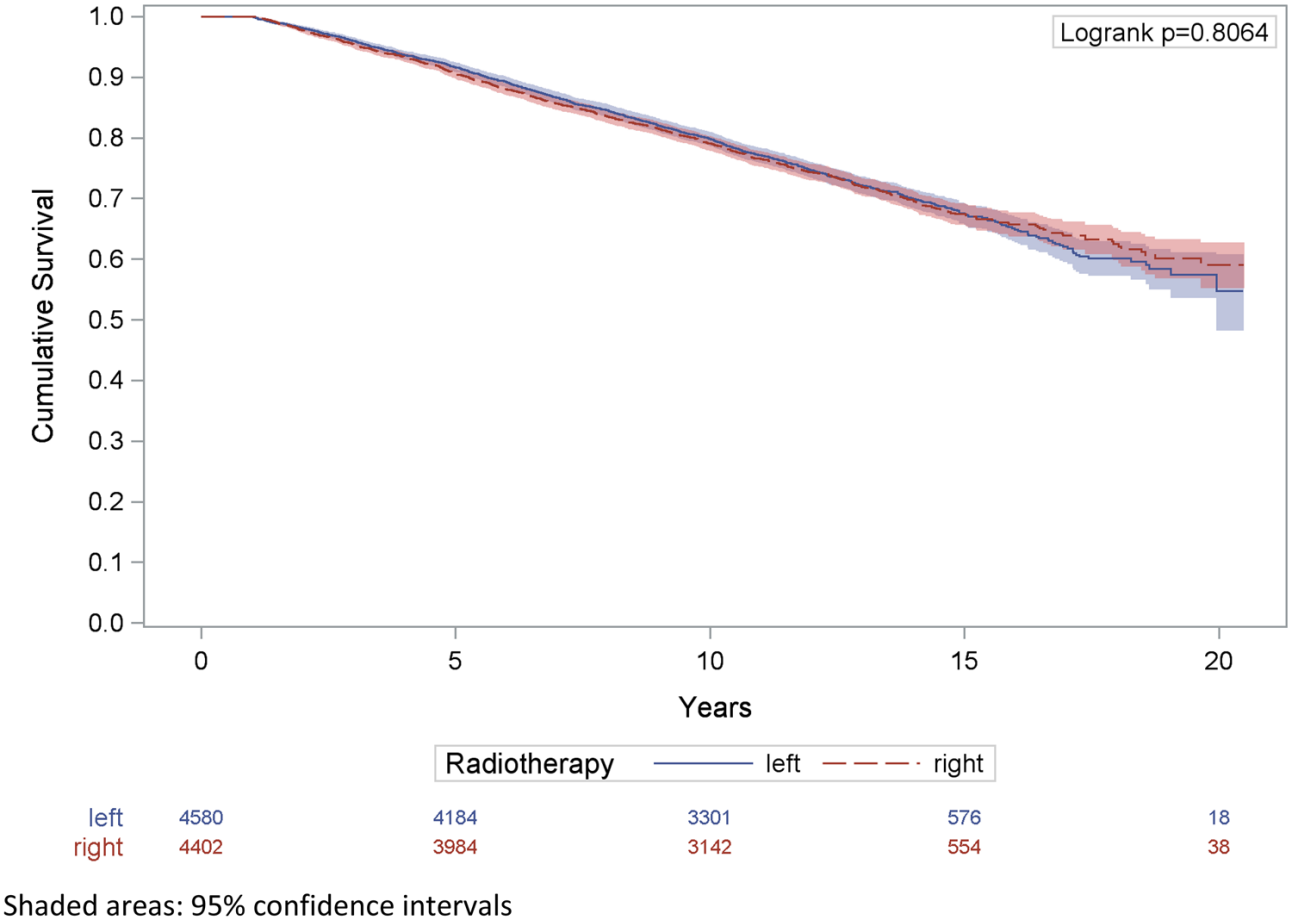
Overall survival of patients with radiotherapy compared by tumor laterality. Shaded areas: 95% confidence intervals

The median survival time among women with RT was 11.4 years (Fig. 1**)**. The overall survival was equivalent between left- and right-sided irradiation at 5-, 10-, and 15-year follow-up increments. The 20-year overall survival was 59% (95%CI 55.2–62.8) in right-sided and 55% (95%CI 48.2–60.8) in left-sided tumors. This result was based on 56 cases only.

### Association between radiotherapy and mortality

**Fig. 2:**
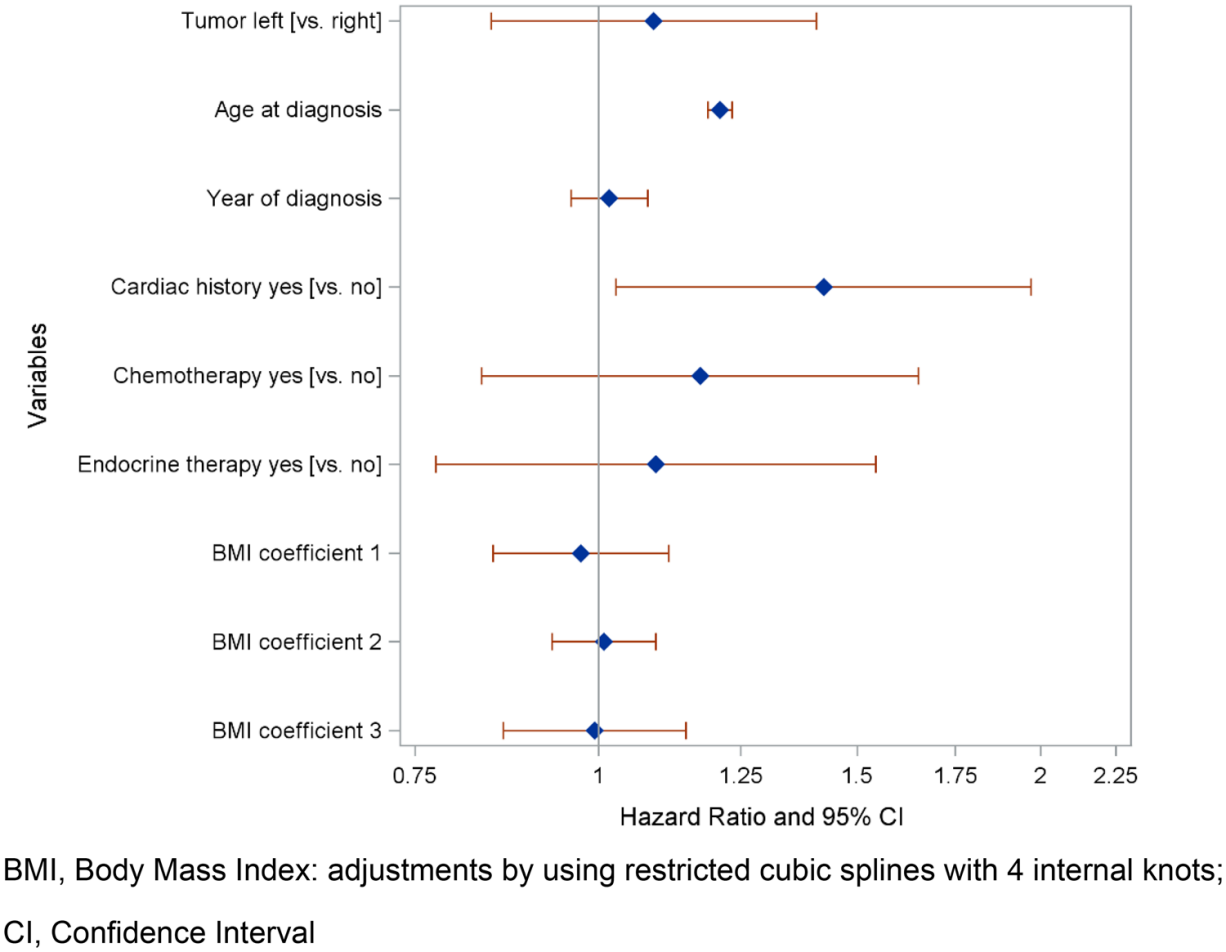
Multivariate cox regression: hazard ratios with 95% confidence intervals for cardiac mortality risk in breast cancer patients with radiotherapy. *BMI* body mass index: adjustments by using restricted cubic splines with four internal knots, *CI* confidence interval

Figure 2 shows the association of laterality with cardiac mortality when controlling for confounders and effect-modifiers. In RT-patients, there was no evidence for an effect of laterality. The HR of cardiac mortality for left-sided vs. right-sided RT was 1.09 (95%CI 0.85–1.41). A history of cardiac disease and higher age at diagnosis were associated with significantly increased cardiac mortality, while chemotherapy was not a significant risk factor. After stratification for the length of follow-up, a statistically not significant increased risk for cardiac mortality due to laterality was detected for an observational period of more than 10 years (HR = 1.35 [95%CI 0.88–2.06]) (Table 3). Sensitivity analyses with censoring at the time of a recurrent event and a complete case analysis did not reveal any different results (SI 2, SI 3). For overall mortality (SI 4), there was no evidence for an effect of laterality in patients treated with RT (HR = 0.97; 95%CI 0.90–1.05). However, in contrast to cardiac mortality, chemotherapy was a significant risk factor for overall mortality (HR = 1.51, 95%CI 1.39–1.65).

**Table 3:** Multivariate cox regression: cardiac mortality risk in breast cancer patients with radiotherapy and stratification for duration of follow-up

			Duration of follow-up			
	All^a^		≥ 1– ≤ 10 years^b^		> 10 years^c^	
	HR	95%CI	HR	95%CI	HR	95%CI
RT, left vs. right	1.09	(0.85–1.41)	0.99	(0.72–1.36)	1.35	(0.88–2.06)
Age at diagnosis	1.21	(1.19–1.23)*	1.15	(1.12–1.18)*	1.21	(1.17–1.26)*
Year of diagnosis	1.02	(0.96–1.08)	0.86	(0.81–0.91)*	1.13	(1.00–1.28)
Cardiac history (yes/no)	1.42	(1.03–1.97)*	0.99	(0.64–1.54)	1.91	(1.16–3.15)*
Chemotherapy (yes/no)	1.17	(0.83–1.65)	0.86	(0.53–1.39)	1.60	(0.97–2.66)
Endocrine therapy (yes/no)	1.09	(0.77–1.54)	0.92	(0.61–1.38)	1.71	(0.90–3.26)
BMI coefficient 1**	0.97	(0.85–1.12)	0.95	(0.81–1.11)	1.02	(0.81–1.30)
BMI coefficient 2	1.01	(0.93–1.09)	1.02	(0.94–1.11)	0.95	(0.82–1.10)
BMI coefficient 3	1.00	(0.86–1.15)	0.97	(0.84–1.13)	1.11	(0.86–1.43)

*BMI* body mass index, 95%CI 95% confidence interval, *HR* hazard ratio, *RT* radiotherapy

*Statistically significant

**BMI coefficient: adjustments for BMI by using restricted cubic splines with four internal knots

^a^Analysis based on 8982 patients: total cohort (*N* = 11,982) with at least 1-year follow-up (11,719), with radio therapy (8982); *n* = 240 cases of cardiac mortality

^b^Analysis based on *N* 2539 individuals with 152 cases of cardiac mortality

^c^Analysis based on *N* 6443 and 88 cases of cardiac mortality

### Morbidity follow-up

In 2014 a total of 5388 questionnaires were received (median follow-up period 8.3 years). The second questionnaire survey had a total of 1.831 responders with a median follow-up of 12.5 years.

Among responders who were treated with RT, at least one incident cardiac event after breast cancer therapy was reported by 497 individuals (from both surveys). Arrhythmia was the most frequent event with an almost equal distribution according to laterality with 12.6% of women with a left-sided tumor (yes 288, no 2,005) and 12.0% of those with a right-sided tumor (yes 261, no 1,920). Myocardial infarction was reported by 2.5% of women (yes 58, no 2,235) with a left-sided tumor and by 1.7% (yes 38, no 2,143) of those with a right-sided tumor.

### Association between radiotherapy and cardiac morbidity

**Fig. 3:**
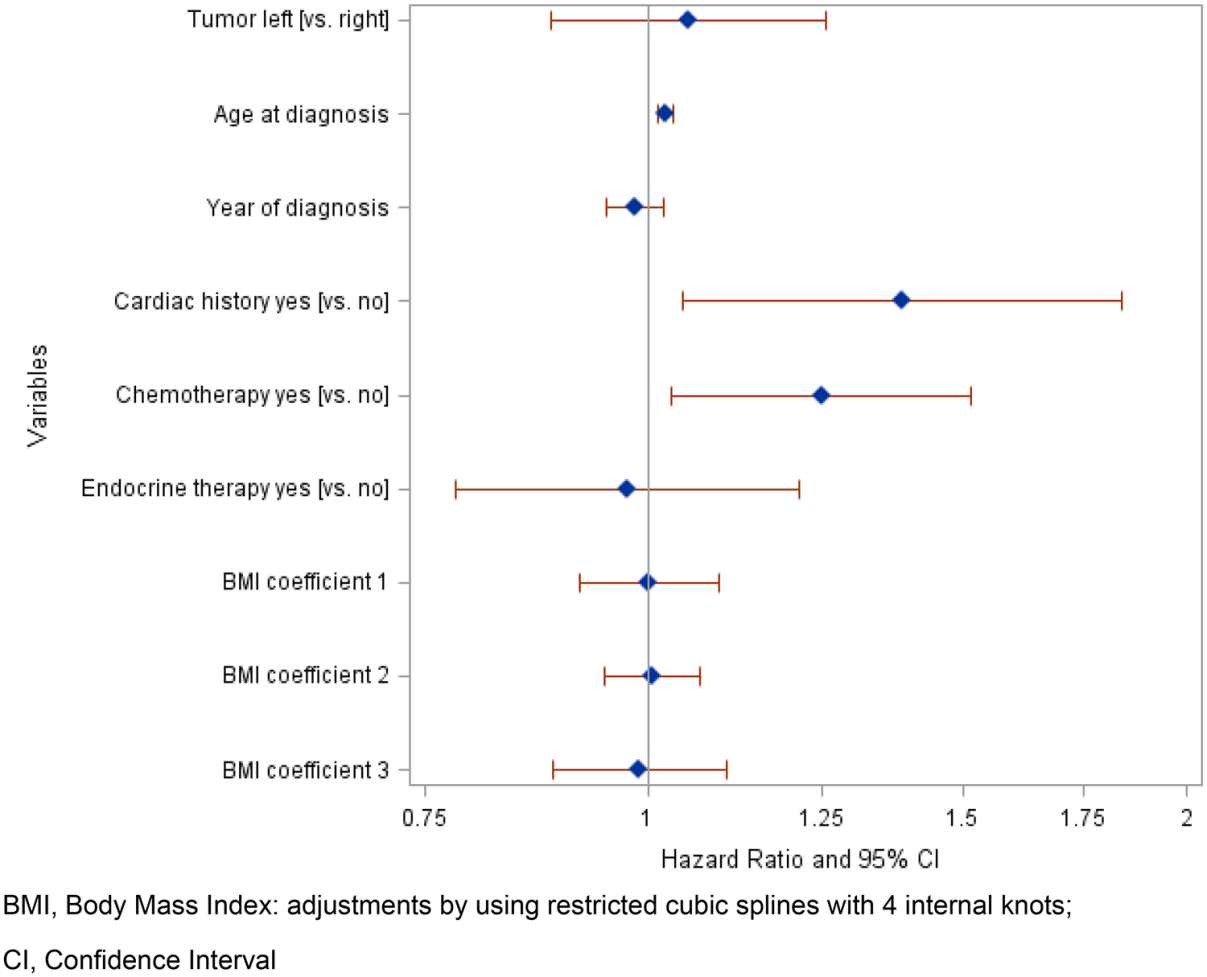
Multivariate cox regression: hazard ratios with 95% confidence intervals for cardiac morbidity in breast cancer patients with radiotherapy. *BMI* body mass index: adjustments by using restricted cubic splines with four internal knots, *CI* confidence interval

Tumor laterality as a surrogate for radiation exposure was not identified as a significant risk factor for any type of cardiac event (Fig. 3). The hazard ratio for left-sided vs. right-sided tumors was 1.05 (95%CI 0.88–1.25). Significant risk factors for cardiac morbidity included a history of cardiac disease at baseline and chemotherapy. Age at diagnosis of breast cancer is a significant risk factor for cardiac morbidity (HR = 1.02; 95%CI 1.01–1.03). Sensitivity analysis did not reveal a significantly higher risk for left-sided RT compared to right-sided RT after more than 10 years of follow-up (Table 4).

**Table 4:** Multivariate cox regression: cardiac morbidity risk in breast cancer patients with radiotherapy and stratification for duration of follow-up

			Duration of follow-up			
	All^a^		≥ 1- ≤ 10 years^b^		> 10 years^c^	
	HR	95%CI	HR	95%CI	HR	95%CI
RT, left vs. right	1.05	(0.88–1.25)	0.94	(0.77–1.15)	1.09	(0.73–1.63)
Age at diagnosis	1.02	(1.01–1.03)*	1.01	(1.00–1.02)*	1.02	(1.00–1.04)*
Year of diagnosis	0.99	(0.95–1.02)	0.77	(0.75–0.80)*	0.88	(0.80–0.97)*
Cardiac history (yes/no)	1.39	(1.05–1.84)*	1.19	(0.87–1.64)	0.96	(0.49–1.90)
Chemotherapy (yes/no)	1.25	(1.03–1.52)*	1.14	(0.92–1.41)	1.20	(0.77–1.86)
Endocrine therapy (yes/no)	0.97	(0.78–1.21)	0.82	(0.64–1.06)	0.98	(0.59–1.60)
BMI coefficient 1**	1.00	(0.92–1.09)	0.98	(0.89–1.08)	1.06	(0.86–1.31)
BMI coefficient 2	1.01	(0.95–1.07)	1.00	(0.95–1.06)	1.00	(0.86–1.16)
BMI coefficient 3	0.99	(0.88–1.11)	1.00	(0.90–1.10)	0.99	(0.75–1.30)

*BMI* body mass index, 95%CI 95% confidence interval, *HR* hazard ratio, *RT* radiotherapy

*Statistically significant

**BMI coefficient: adjustments for BMI by using restricted cubic splines with four internal knots

^a^Analysis based on 4379 patients: total sub-cohort (5388) with at least 1-year follow-up (5280), who received radio therapy (4379); *n* = 497 cases of incident cardiac morbidity

^b^Analysis based on *N* 883 individuals with 402 cases of cardiac morbidity

^c^Analysis based on *N* 3496 and 95 cases of cardiac morbidity

## Discussion

The ESCaRa-Study investigated the relationship between 3D-conformal RT and long-term cardiac mortality and morbidity risk in a cohort of 11,982 patients treated between 1998 and 2008 in Germany. We evaluated the impact of left-sided vs. right-sided radiation as a proxy measure for the exposure of the heart to ionizing radiation.

### Main results

A multivariate analysis revealed no evidence for an effect of laterality on cardiac mortality in 9057 women treated with RT (HR, left-sided vs. right-sided tumor 1.09; 95%CI 0.85–1.41) after a median follow-up time of 11.1 years. Tumor laterality was not identified as a significant risk factor for incident cardiac morbidity (HR = 1.05; 95%CI 0.88–1.25).

### Comparison to earlier studies

A Surveillance, Epidemiology and End Results Study (SEER) evaluated the difference in cardiac mortality among breast cancer patients who received RT. For women irradiated with contemporary techniques and in more recent years (1993 +), there was little evidence for an increased cardiac mortality considering laterality as a proxy for the radiation exposure of the heart [26, 27]. This finding was supported by Li et al. [28] who analyzed SEER-data of more than 168,000 breast cancer patients diagnosed between 2000 and 2008. After a median follow-up time of 8.8 years, no association between tumor laterality and cardiac-related mortality was found. Investigations of smaller cohorts agree with these observations [29–31], suggesting that newer RT techniques may not increase the cardiac mortality risk. A large cohort study with a total of 1,934,248 breast cancer patients from 22 countries evaluated the difference in cardiac mortality among left and right breast irradiated women [9]. Considering all women diagnosed 1990–2002 the cardiac mortality rate ratio left vs. right was not significantly different from unity (Relative Risk = 0.98; 95%CI 0.93–1.02). The mean length of follow-up was 6.7 years which might explain the lack of evidence [9]. Overall, our results on cardiac mortality and modern RT in German breast cancer patients are consistent with the results of earlier studies. However, even a median follow-up period of 11.1 years might not to be sufficient to detect a possible excess mortality.

Studies on tumor laterality as a potential risk factor for cardiac morbidity in patients treated after 1990 show inconclusive results. Studies in older women aged 65 + found no significant increase in heart disease [30, 32, 33]. Registry-based studies included the full age range of breast cancer patients [34–36] and indicated a moderately increased risk, based on large sample sizes. Among Danish patients irradiated with modern RT (2008–2016), the incidence rate ratio for cardiac event (left vs. right-sided) was 0.90 (95%CI 0.69–1.16) after a median follow-up of 6.9 years [37]. The ESCaRa-Study failed to demonstrate an increased risk of cardiac morbidity following left breast RT in German patients, even after a follow-up period of 12 years.

### Confounding and interaction

Rehammar et al. [36] have shown that anthracycline-based chemotherapy further raises the risk of heart disease in left-sided compared to right-sided irradiation. In the ESCaRa-cohort only 50% of the irradiated patients received adjuvant chemotherapy. We found a risk increase for cardiac outcomes related to chemotherapy, but without statistical significance for cardiac mortality. However, chemotherapy was a statistically significant risk factor for all-cause mortality (HR = 1.51; 95%CI 1.39–1.65). This observation might be related to confounding by indication and competing risks: a severe breast cancer disease associated with a poorer prognosis indicates the application of chemotherapy. An unfavorable cancer staging and worse survival might remove a subject from being at risk for long-term cardiovascular disease or cardiovascular death.

Obesity is one of the most frequently reported risk factor for cardiovascular disease [38] and an established risk factor for postmenopausal breast cancer [39].The presented cohort did not identify BMI as significant cardiac risk factors. While BMI was not a statistically significant risk factor for cardiac mortality in multivariate analysis, BMI < 20 and > 23 were descriptively associated with higher risk which flattened at BMI > 28 (SI 5).

### Strengths and limitations

The ESCaRa-Study permitted an effective mortality follow-up (loss to follow-up 1.1%) and a comprehensive ascertainment of cause of death (completeness 97%). The available information on morbidity endpoints in the present study comes from a questionnaire survey with a moderate to high response rate (58.9% first survey 2014; 80% follow-up survey 2019). Further strengths of the present study include the extensive clinical documentation of breast cancer patients treated in certified breast centers. By including a comparably large number of observed cardiac events (240 cases of cardiac mortality; 497 cases of cardiac morbidity), the statistical model could adjust for several cardiac risk factors that are potential modifiers for the effect of tumor laterality on risk for late cardiac events.

The median mortality follow-up time in the ESCaRa-Study was 11.1 years, the median follow-up period for morbidity endpoints was 12.4 years. No statistically significant evidence for an increased risk of cardiac mortality or cardiac morbidity in irradiated women was found. It is possible that the follow-up period was too short to detect long-term adverse health hazards of RT. The risk for coronary heart disease started within the first decade, whereas the risk for cardiac death started from the second decade after RT [7].

When deriving inferences from questionnaire survey data, it is important to consider any self-selection bias. The second survey (2019) focused on responders of the first survey, who gave permission for being contacted again. This diminished the targeted sample and may have introduced selection of healthy survivors. Survey participants, especially those who participated in both surveys, may have been motivated by a higher interest in late cardiac effects which might be associated with a healthier life-style compared to non-responders. Furthermore, self-reported events may be prone to information bias due to restrictions in memory, a lack of fully understanding medical diagnoses, or selective reporting [40]. However, a validation study revealed moderate to fair agreement between self-reported events compared with medical records from general practitioners [41].

Finally, laterality is a crude proxy measure for the exposure of radiation to the heart. In terms of an increased risk of heart disease many years after irradiation, scientific efforts are needed to investigate susceptible regions and structures of the heart, to assess exact cardiac doses, and to ascertain a possible threshold and dose–response relationship. In the future, it is essential to individualize treatment according to tumor characteristics, treatment modalities (endocrine and immunotherapy) and patients characteristics [42, 43].

## Conclusions

The population-based ESCaRa-study offers continued support for the efficacy of modern breast cancer treatment. We found no indication that radiotherapy for left-sided breast cancer in German patients is a strong limiting factor for survival in the first decade after breast cancer therapy.

## Supplementary Information

Below is the link to the electronic supplementary material.

Supplementary file1 (DOCX 45 KB)

## Data Availability

Raw data compliant with the institutional confidentiality policies can be available upon request from the corresponding author.

## Abbreviations

BMI: Body mass index
CIS: Carcinoma in situ
CI: Confidence interval
Gy: Gray
ICD: International classification of diseases
HR: Hazard ratio
NYHA: New York heart association
RT: Radiotherapy
SI: Supplementary information
