# Supplementary material for: Cardiac late effects after modern 3D-conformal radiotherapy in breast cancer patients: a retrospective cohort study in Germany (ESCaRa)
Source: Breast Cancer Res Treat. 2021 Oct 9;191(1):147–57. doi: 10.1007/s10549-021-06412-3 (PMC8758608; doi:10.1007/s10549-021-06412-3)
Supplement: Supplementary file 1 — Supplementary file1 (DOCX 45 KB) [file 10549_2021_6412_MOESM1_ESM.docx]

Cardiac late effects after modern radiotherapy in breast cancer patients - a retrospective cohort study in Germany (ESCaRa)

Hiltrud Merzenich, Dan Baaken, Marcus Schmidt, Inga Bekes, Lukas Schwentner, Wolfgang Janni, Achim Woeckel, Detlef Bartkowiak, Thomas Wiegel, Maria Blettner, Daniel Wollschläger, Heinz Schmidberger

Corresponding author: Hiltrud Merzenich, hmerzeni@uni-mainz.de

**BREA-D-21-00912**

**Supplementary Information, SI 1**

**Short questionnaire on cardiac illness and on cardiovascular risk factors**

| **Did a physician ever diagnosed one of the following diseases?**  (multiple answers possible**)** |  |  | **If yes, when did you get the diagnosis for the first time?**  (Please, indicate age **or** calendar year) | |
| --- | --- | --- | --- | --- |
|  | No | Yes | Age | Calendar Year |
| Myocardial infarction | □ | □ | \|__\|\|__\|  Year | \|__\|__\|__\|__\|  (Year) |
| Angina pectoris | □ | □ | \|__\|\|__\|  Year | \|__\|__\|__\|__\|  (Year) |
| Congestive heart failure  (ischemic heart disease) | □ | □ | \|__\|\|__\|  Year | \|__\|__\|__\|__\|  (Year) |
| Arrythmia of the heart | □ | □ | \|__\|\|__\|  Year | \|__\|__\|__\|__\|  (Year) |
| Valvular heart disease | □ | □ | \|__\|\|__\|  Year | \|__\|__\|__\|__\|  (Year) |
| Stroke | □ | □ | \|__\|\|__\|  Year | \|__\|__\|__\|__\|  (Year) |
| Diabetes mellitus | □ | □ | \|__\|\|__\|  Year | \|__\|__\|__\|__\|  (Year) |
| Hypertension | □ | □ | \|__\|\|__\|  Year | \|__\|__\|__\|__\|  (Year) |
| Adiposits, overweight | □ | □ | \|__\|\|__\|  Year | \|__\|__\|__\|__\|  (Year) |
| Increased blood cholesterol,  increased blood lipids | □ | □ | \|__\|\|__\|  Year | \|__\|__\|__\|__\|  (Year) |
| Chronic Lung Disease  (Asthma, Bronchitis, Chronic obstructive  pulmonary disease | □ | □ | \|__\|\|__\|  Year | \|__\|__\|__\|__\|  (Year) |
| Chronic kidney disease | □ | □ | \|__\|\|__\|  Year | \|__\|__\|__\|__\|  (Year) |
| Thyroid functional disease | □ | □ | \|__\|\|__\|  Year | \|__\|__\|__\|__\|  (Year) |

Did you have a pacemaker? No, don’t know, yes, if yes-when (calendar year)?

Did you have a stent implantation? No, don’t know, yes, if yes-when (calendar year)?

Was a balloon dilatation done? No, don’t know, yes, if yes-when (calendar year)?

Was a pass surgery done? No, don’t know, yes, if yes-when (calendar year)?

**Supplementary Information, SI 2**

**Multivariate Cox Regression: cardiac mortality risk in breast cancer patients with radiotherapy and stratified for duration of follow-up with follow-up censored at the time of diagnosis of a recurrent event**

|  |  | | **Duration of follow-up** | | | |
| --- | --- | --- | --- | --- | --- | --- |
|  | **All^a^** | | **>=1-<=10 years^b^** | | **>10 years^c^** | |
|  | HR | 95% CI | HR | 95% CI | HR | 95% CI |
| RT, left vs. right | 1.09 | (0.84-1.41) | 0.97 | (0.70-1.34) | 1.41 | (0.91-2.17) |
| Age at diagnosis | 1.21 | (1.19-1.23)***** | 1.15 | (1.12-1.18)***** | 1.21 | (1.17-1.25)***** |
| Year of diagnosis | 1.02 | (0.96-1.08) | 0.86 | (0.81-0.91)***** | 1.15 | (1.01-1.30) |
| Cardiac history (yes/no) | 1.42 | (1.03-1.97)***** | 1.00 | (0.64-1.56) | 1.79 | (1.07-2.99)***** |
| Chemotherapy (yes/no) | 1.16 | (0.82-1.65) | 0.90 | (0.56-1.45) | 1.50 | (0.89-2.52) |
| Endocrine therapy (yes/no) | 1.10 | (0.78-1.56) | 0.94 | (0.62-1.42) | 1.62 | (0.85-3.11) |
| BMI coefficient 1** | 0.97 | (0.84-1.11) | 0.95 | (0.81-1.11) | 1.02 | (0.81-1.30) |
| BMI coefficient 2 | 1.01 | (0.93-1.10) | 1.02 | (0.94-1.12) | 0.94 | (0.82-1.09) |
| BMI coefficient 3 | 0.99 | (0.86-1.15) | 0.97 | (0.83-1.13) | 1.12 | (0.86-1.45) |

Abbreviations: BMI, Body Mass Index; 95% CI, 95% Confidence Interval; HR, Hazard Ratio; RT, radiotherapy

^a^Analysis based on 8,856 and n = 235 cases of cardiac mortality

^b^Analysis based on N 2,452 individuals with 150 cases of cardiac mortality;

^c^Analysis based on N 6,404 and 85 cases of cardiac mortality;

***** statistically significant

** BMI coefficient: adjustments for BMI by using restricted cubic splines with 4 internal knots

**Supplementary Information, SI 3**

**Multivariate Cox Regression: cardiac mortality risk in breast cancer patients with radiotherapy and stratified for duration of follow-up, based on a complete case-analysis**

|  |  | | **Duration of follow-up** | | | |
| --- | --- | --- | --- | --- | --- | --- |
|  | **All^a^** | | **>=1-<=10 years^b^** | | **>10 years^c^** | |
|  | HR | 95% CI | HR | 95% CI | HR | 95% CI |
| RT, left vs. right | 1.10 | (0.84-1.45) | 1.04 | (0.73-1.47) | 1.33 | (0.85-2.07) |
| Age at diagnosis | 1.22 | (1.20-1.25)***** | 1.16 | (1.13-1.18)***** | 1.24 | (1.20-1.29)***** |
| Year of diagnosis | 1.01 | (0.95-1.08) | 0.85 | (0.80-0.91)***** | 1.12 | (0.98-1.27) |
| Cardiac history (yes/no) | 1.42 | (1.00-2.02)***** | 0.79 | (0.48-1.30) | 2.18 | (1.29-3.69)***** |
| Chemotherapy (yes/no) | 1.20 | (0.83-1.72) | 0.88 | (0.53-1.46) | 1.70 | (1.00-2.89) |
| Endocrine therapy (yes/no) | 1.10 | (0.79-1.64) | 1.01 | (0.66-1.56) | 1.69 | (0.88-3.26) |
| BMI coefficient 1** | 0.97 | (0.85-1.11) | 0.94 | (0.81-1.10) | 1.02 | (0.81-1.29) |
| BMI coefficient 2 | 1.01 | (0.96-1.09) | 1.02 | (0.94-1.11) | 0.95 | (0.82-1.09) |
| BMI coefficient 3 | 1.00 | (0.87-1.16) | 0.98 | (0.84-1.13) | 1.12 | (0.87-1.44) |

Abbreviations: BMI, Body Mass Index; 95% CI, 95% Confidence Interval; HR, Hazard Ratio; RT, radiotherapy

^a^ Analysis based on 8,007 and n = 214 cases of cardiac mortality

^b^ Analysis based on N 2,221 individuals with 133 cases of cardiac mortality;

^c^ Analysis based on N 5,786 and 81 cases of cardiac mortality;

***** statistically significant

** BMI coefficient: adjustments for BMI by using restricted cubic splines with 4 internal knots

**Supplementary Information, SI 4**

**Multivariate Cox Regression: overall mortality risk in breast cancer patients with radiotherapy for the EScaRa-cohort and stratified for duration of follow-up**

|  |  | | **Duration of follow-up** | | | |
| --- | --- | --- | --- | --- | --- | --- |
|  | **All^a^** | | **>=1-<=10 years^b^** | | **>10 years^c^** | |
|  | HR | 95% CI | HR | 95% CI | HR | 95% CI |
| RT, left vs. right | 0.97 | (0.90-1.05) | 0.93 | (0.85-1.02) | 1.07 | (0.92-1.26) |
| Age at diagnosis | 1.06 | (1.06-1.07)***** | 1.03 | (1.02-1.03)***** | 1.09 | (1.08-1.10)***** |
| Year of diagnosis | 0.97 | (0.96-0.99)***** | 0.83 | (0.81-0.84)***** | 0.99 | (0.95-1.03) |
| Cardiac history (yes/no) | 1.21 | (1.07-1.36)***** | 0.87 | (0.75-1.01) | 1.22 | (0.98-1.52)***** |
| Chemotherapy (yes/no) | 1.51 | (1.39-1.65)***** | 1.52 | (1.37-1.69)***** | 1.31 | (1.10-1.56)***** |
| Endocrine therapy (yes/no) | 0.77 | (0.70-0.85)***** | 0.71 | (0.64-0.79)***** | 1.21 | (0.98-1.50) |
| BMI coefficient 1** | 0.96 | (0.92-1.00) | 1.01 | (0.97-1.05) | 0.95 | (0.88-1.02) |
| BMI coefficient 2 | 1.02 | (1.00-1.05) | 1.00 | (0.97-1.02) | 1.02 | (0.97-1.07) |
| BMI coefficient 3 | 0.97 | (0.93-1.01) | 1.01 | (0.97-1.05) | 0.98 | (0.90-1.07) |

Abbreviations: BMI, Body Mass Index; 95% CI, 95% Confidence Interval; HR, Hazard Ratio; RT, radiotherapy

^a^Analysis based on 8,982 patients: total cohort (N=11,982) with at least one-year follow-up (11,719), with radiotherapy (8,982); n = 2460 cases of death

^b^Analysis based on N 2539 and 1833 cases of death

^c^Analysis based on N 6443 and 627 cases of death

***** statistically significant

** BMI coefficient: adjustments for BMI by using restricted cubic splines with 4 internal knots

**Supplementary Information, SI 5**

**Marginal plot of BMI at time of breast cancer diagnosis using restricted cubic splines with 4 internal knots plotted against Hazard Ratios for cardiac mortality risk in breast cancer patients with radiotherapy (all other covariates were held constant)**


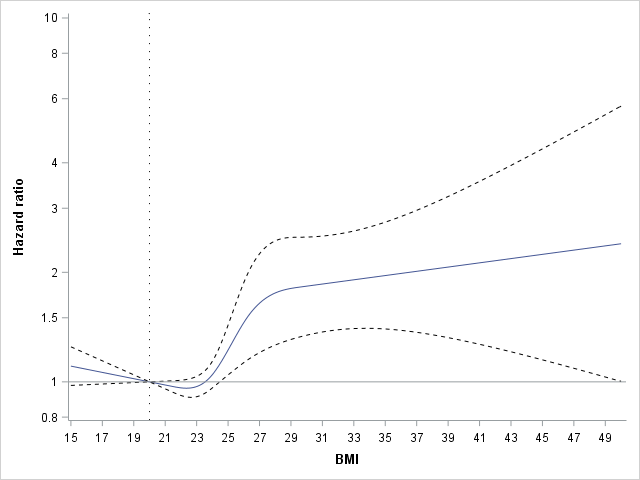


Abbreviation: BMI, Body Mass Index;

Dashed lines: 95% confidence intervals
